# Bilateral sphenoid sinus mucocele observed in Yaoundé-Cameroon: a case report

**DOI:** 10.11604/pamj.2021.39.198.22886

**Published:** 2021-07-13

**Authors:** Antoine Siafa Bola, Yves Christian Nkouo Andjock, Emmanuel Nchinda Choffor, Eliane Nanci, Ignatius Ngene Esene, Francois Djomou

**Affiliations:** 1 Department of Ophthalmology-Otolaryngology-Stomatology, Faculty of Medicine and Biomedical Sciences, University of Yaoundé I, Yaoundé, Cameroon,; 2 Department of Surgery, Faculty of Health Sciences, Buea, Cameroon,; 3 Neurosurgery Division, Faculty of Health Sciences, University of Bamenda, Bambili, Cameroon

**Keywords:** Sphenoid sinus mucocele, sinus cavity, marsupialisation, Yaoundé, case report

## Abstract

A mucocele is a cystic and expansive lesion of the sinus cavities. It is uncommon in the sphenoid sinus and its management is quite challenging especially in low to middle income countries like Cameroon. A 43-years-old female was referred to us by a neurologist for chronic headache and visual disturbances. The pain was unresponsive to analgesics. Physical examination was non-remarkable and a head CT scan realized showed a cyst-like lesion in the sphenoid sinus cavity. Surgical endoscopic treatment was proposed and realized with basic endoscopic instruments, consisting of opening the cavity with drainage of the mucocele. A large opening was made on the anterior wall of the sphenoid sinus, in order to ensure continuous drainage and prevent a recurrence. Sphenoid sinus mucocele is a rare condition, and its diagnosis can be difficult. Confirmation requires specific imaging and treatment is presently well established, but it can be managed with basic tools.

## Introduction

A mucocele is defined as a benign expansive pseudo-cyst of the sinus cavities. It is a rare condition made up of a wall consisting of a modified mucosal lining and a viscous liquid content that is generally aseptic [1]. This lesion is usually progressive with marked loco-regional aggressiveness [1]. Mucoceles are mostly discovered incidentally, or as a result of their effect on surrounding organs. They frequently develop in the frontal and ethmoid sinus cavities [1]. The first reported case of sphenoid mucocele was described by Berg in 1889 [2]. Since then, less than 200 cases have been reported across the world. Little or no papers have reported cases of sphenoid mucoceles in sub-Saharan Africa. We report a case of bilateral sphenoid mucocele observed at the Yaoundé University Teaching Hospital, Cameroon. We describe the diagnostic methods used and management approach in our setting. This is the first case of sphenoid sinus mucocele reported in Cameroon, to the best of our knowledge.

## Patient and observation

**Patient information:** a 43-year-old female, with no relevant past medical history, was addressed to our service by a neurologist for an opinion on chronic severe retro-orbital headache and blurred vision ongoing for about seven months, she also complained of chronic intermittent rhinorrhoea. The headache was unresponsive to chronic non-opioid analgesic use.

**Clinical findings:** physical examination done at our unit showed stable vital signs (blood pressure: 126/87mmHg, pulse rate 69 beats per minute), and a normal nasal cavity examination. Flexible nasopharyngoscopy was normal. The rest of the ear-nose-throat and head and neck examination was non-remarkable.

**Figure 1:**
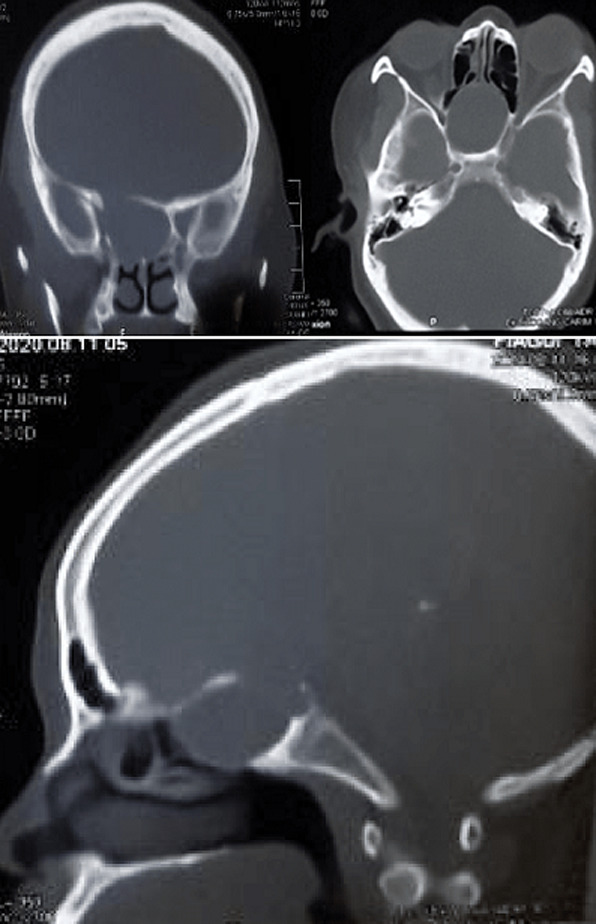
CT scan of the head, showing a roundish isodense lesion occupying the whole sphenoid sinus cavity with erosion of the roof

**Diagnosis assessment:** a head CT-Scan was requested and identified a roundish isodense lesion occupying both sinus cavities with absence of the intersinus septum. The lesion eroded the walls of the sphenoid sinus, with partial lysis of the superior wall of the cavity (Figure 1).

**Diagnosis:** a diagnosis of sphenoid mucocele was suspected based on these findings. An MRI was then requested but not done due to financial limitations.

**Figure 2:**
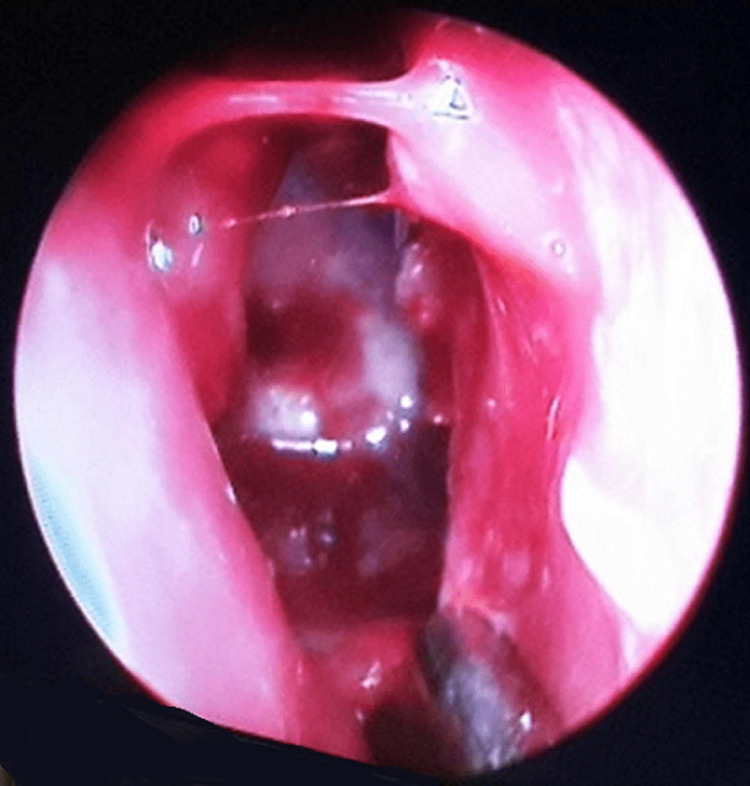
endoscopic view, showing an opening created through the sphenoid anterior ostium by enlarging it

**Therapeutic interventions:** the patient was managed surgically using basic endoscopic sinus forceps, comprising of an anterior endoscopic sphenoidotomy under general anaesthesia (Figure 2), followed by marsupialisation. A wide opening of the anterior sinus wall permitted drainage of a viscous, yellowish liquid, suggestive of superinfection, with disappearance of the intersinusal septum and fusion of both cavities confirming the diagnosis of sphenoid mucocele.

**Figure 3:**
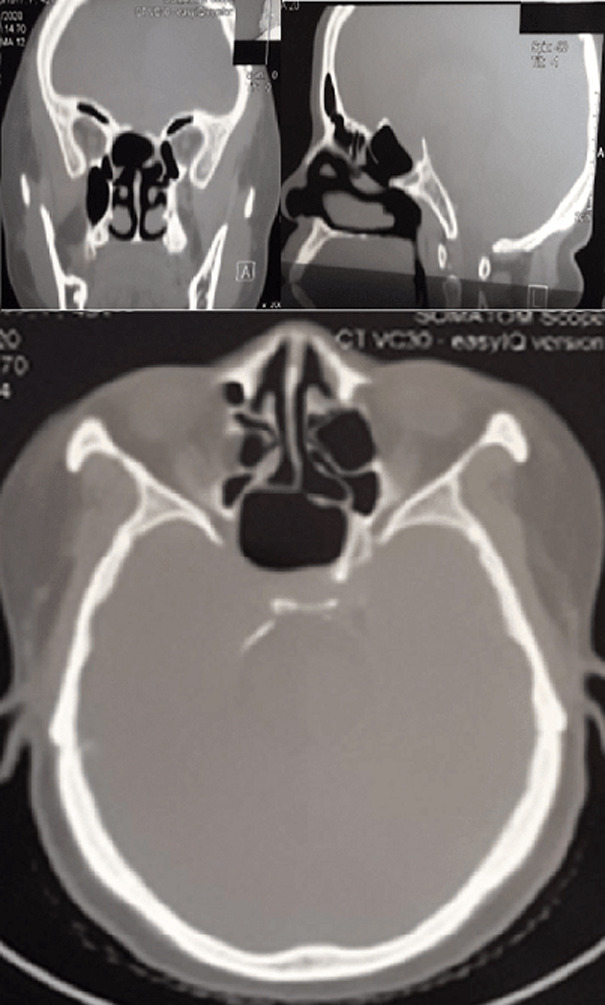
CT scan of the head, one month after the endoscopic surgical treatment, there is perfect aeration of the sphenoid sinus

**Follow-up and outcome of intervention:** post-operative follow-up was non-remarkable. A rigid endoscopic review was carried out three days and one month after surgery, and showed good evolution. The symptoms disappeared a few days after surgery and there has not been any recurrence reported (Figure 3).

**Patient´s perspective and informed consent:** the patient felt completely relieved one month after the surgery and was very happy to return to her daily activities. She gave her full written informed consent for the publication of this case report.

## Discussion

A mucocele is literally a mucus sac, comprising of an epithelial lining filled with mucus. This sac occupies one or more sinus cavities, with propensity toward expansion [3]. The cause of this lesion can be traumatic or iatrogenic. It is a rare condition that is most common in the frontal and ethmoid sinuses [4], exceptionally in the sphenoid sinus. Diagnosis is evoked based on clinical elements like retro-orbital headache, blurred vision, explained by pressure applied by the sac on surrounding organs; meninges, optical nerve. A head CT-Scan focussing on the sinus cavities generally suspects the diagnosis, permitting visualisation of a tissue density lesion with regular borders with the potential to cause bone erosion and deformation, as was the case in our patient (Figure 1). On MRI, mucoceles present variable signals on T1-weighted images, but hyperintense signals on T2. These characteristics are unique to mucoceles and permit differentiation from other sinus cavity lesions like polyps and other potentially associated tumours [4].

Sphenoid mucoceles represent only 1-2% of all mucocele locations [5]. They usually occur around the fourth decade of life (our patient was 43 years old), with no predilection for sex [6,7]. Differential diagnoses on CT-Scan include pituitary adenoma, epidermoid cyst, arachnoid cyst, craniopharyngioma, cystic glioma of the optic nerve [8]. MRI is key to confirm the diagnosis, permitting the identification of a cystic lesion originating from the sphenoid sinus [8]. In our patient, only a CT-scan was done due to financial limitations. However, a good CT-Scan with diligent analysis of all views could be sufficient in resource-limited settings.

Management of sphenoid mucoceles is surgical, preferably by video-assisted endoscopy. The sinus cavity is accessed via the anterior wall, realising a sphenoidotomy, followed by drainage of the cavity content. Next, an enlargement of the medial and inferior aspects of the natural drainage orifice of the sinus, or of the artificially creating orifice, is realised. This technique permits to maintain the cavity open while allowing continuity of the sinus mucosa with that of the nasal cavities (marsupialisation), reducing the risk of recurrence [9]. This procedure, which is relatively easy to master, was used in our patient using basic endoscopic sinus surgery equipment.

The prognosis of sinus cavity mucoceles is generally good when diagnosis and management are prompt. Neurological symptoms mostly regress when treatment is timely

## Conclusion

The sphenoid sinus mucocele is a rare condition for which diagnosis can be challenging, especially in resource-limited settings. A CT-scan is sufficient enough in the presence of suggestive clinical signs and symptoms to make the diagnosis. The management may only require basic tools of functional endoscopic surgery in our context.

## References

[1] Marrakchi J, Hachicha H, Bechraoui R, Chaabouni M, Zainine R, Ben amor M (2014). Les mucocèles nasosinusiennes : étiopathogénie. Anales françaises d´oto-rhino-laryngologie et de pathologie cervico-faciale.

[2] Berg J (1889). Bidrag till Kannendomen om sjirkdomarna I nasans bihalor samt till laran om cerebrospinal-vatskas flytning urnasam. Nord Med.

[3] Aaron Friedman, Pete Batra S, Samer Fakhri, Martin Citardi J, Donald Lanza C (2005). Isolated sphenoid sinus disease: etiology and management. Otolaryngol Head Neck Surg.

[4] Gavioli C, Grasso DL, Carini F, Amoroso C, Pastore A (2002). Mucoceles of the frontal sinus. Clinical and therapeutical considerations. Minerva Stomatol.

[5] Llyod G, Lund VJ, Savy L, Howard D (2002). Optimum imaging for mucocèle. J Laryngol Otol.

[6] Sethi DS, Lan DP, Chan C (1997). Sphenoid sinus mucocèle presenting with isolated oculomotor nerve palsy. J Laryngol Otol.

[7] Iqbal J, Kanaan I, Ahmed M, Al Homsi M (1998). Neurosurgical aspects of sphenoid sinus mucocèle. Br J Neurosurg.

[8] Kataria R, Gupta S, Chopra S, Bagaria H, Sinha VD (2012). Mucocele of the sphenoid sinus: a rare cause of reversible third nerve palsy. Ann Indian Acad Neurol.

[9] Lee LA, Huang CC, Lee TJ (2004). Prolonged visual disturbance secondary to isolated sphenoid sinus disease. Laryngoscope.

